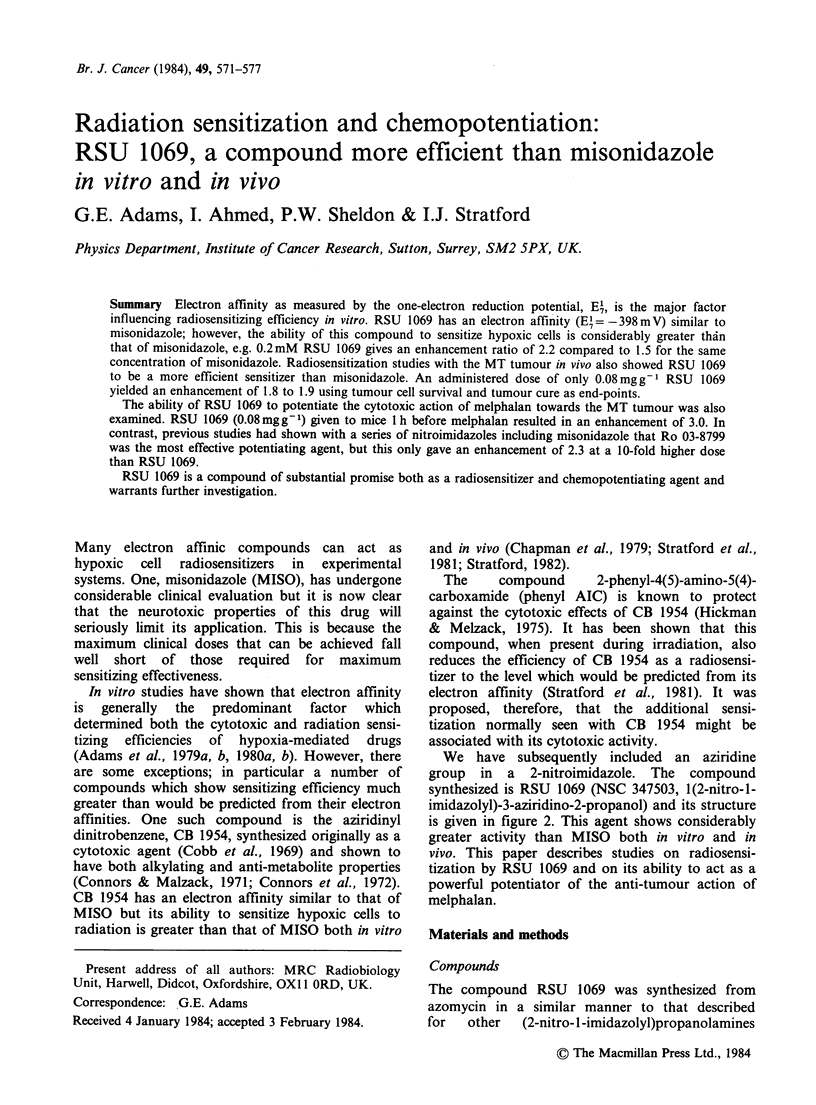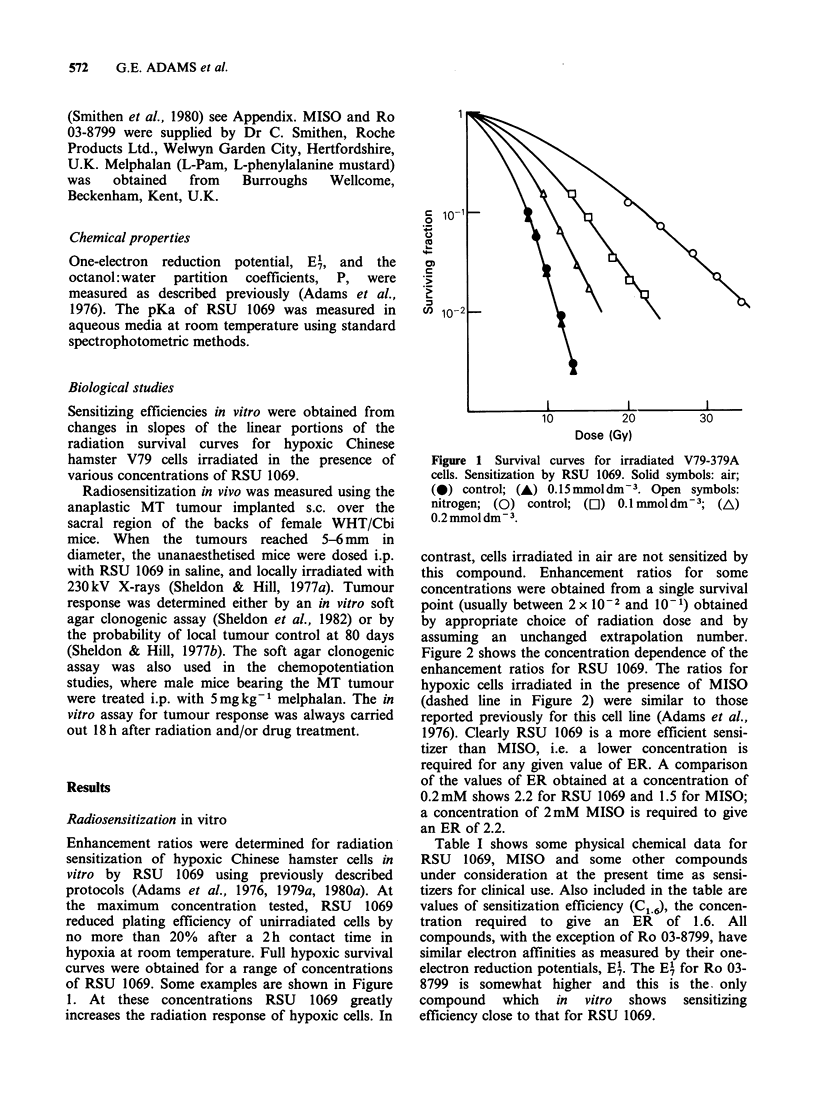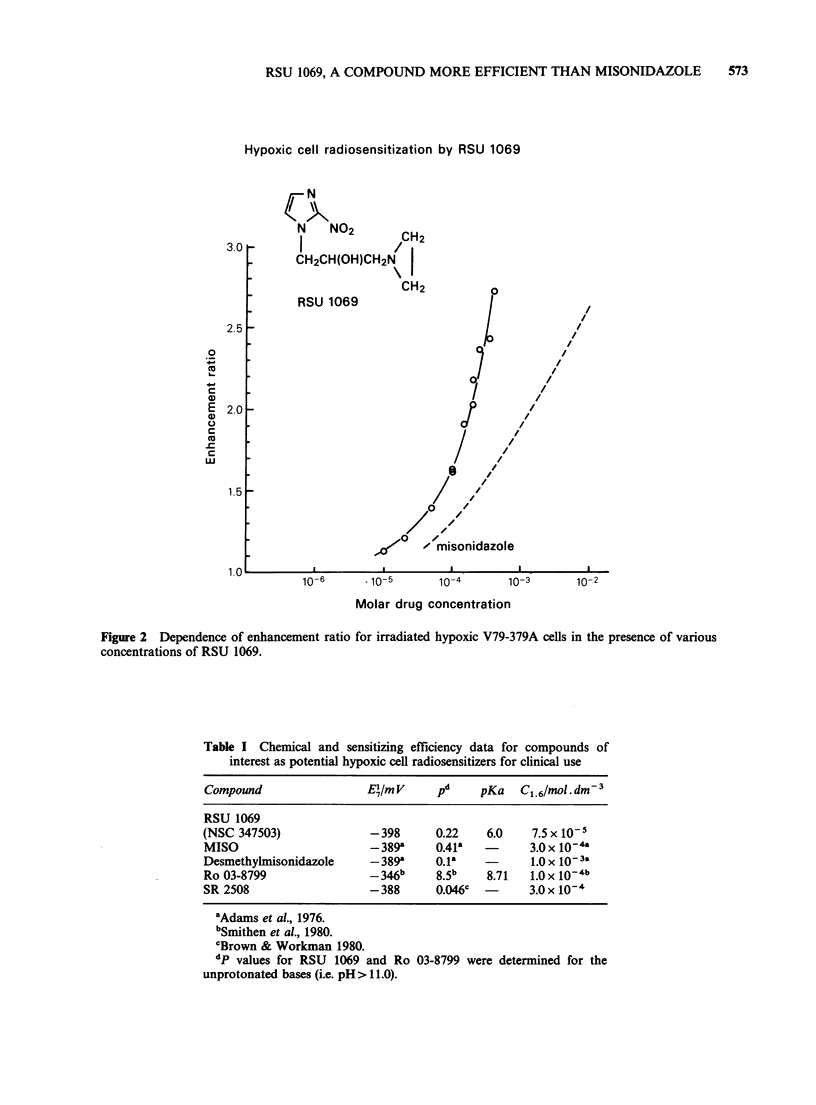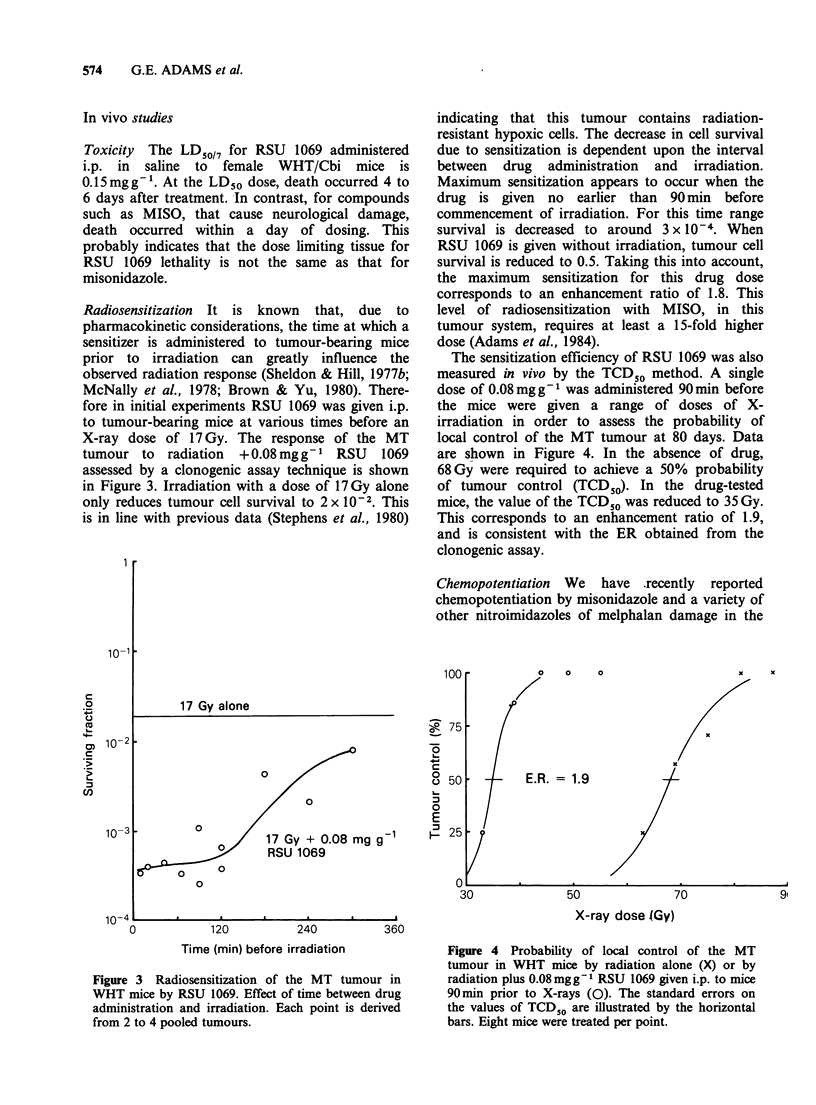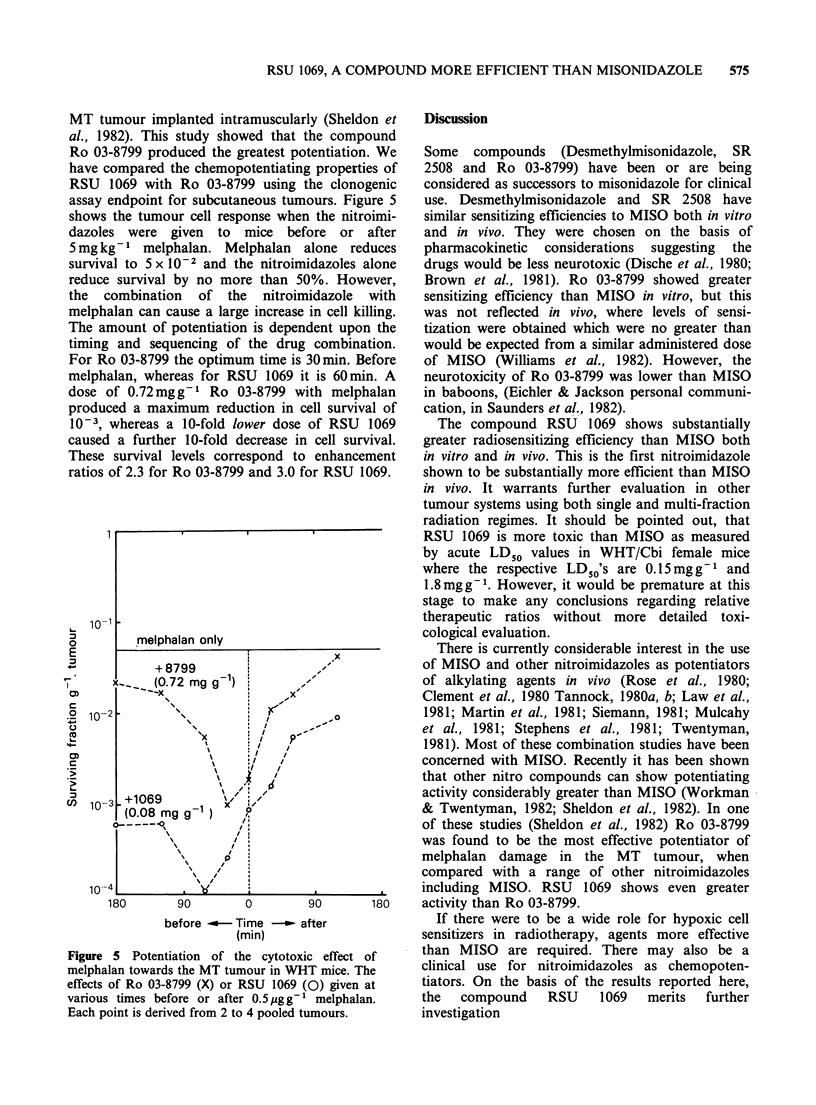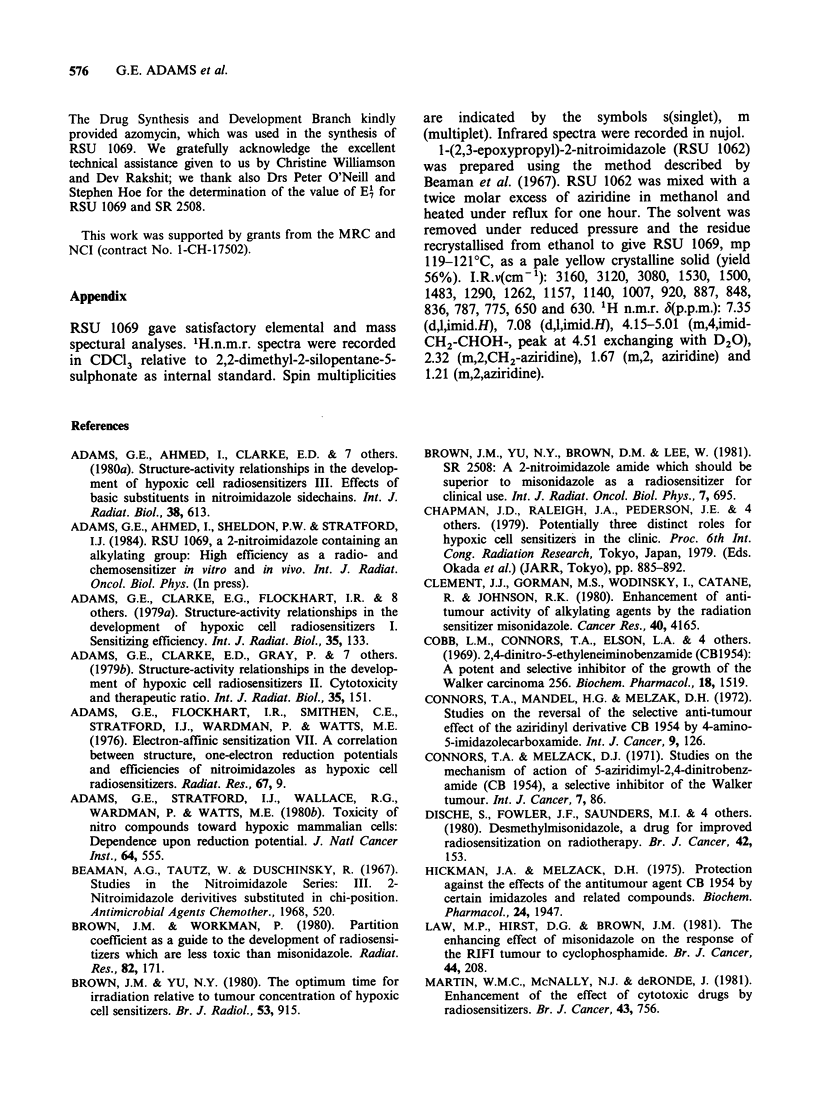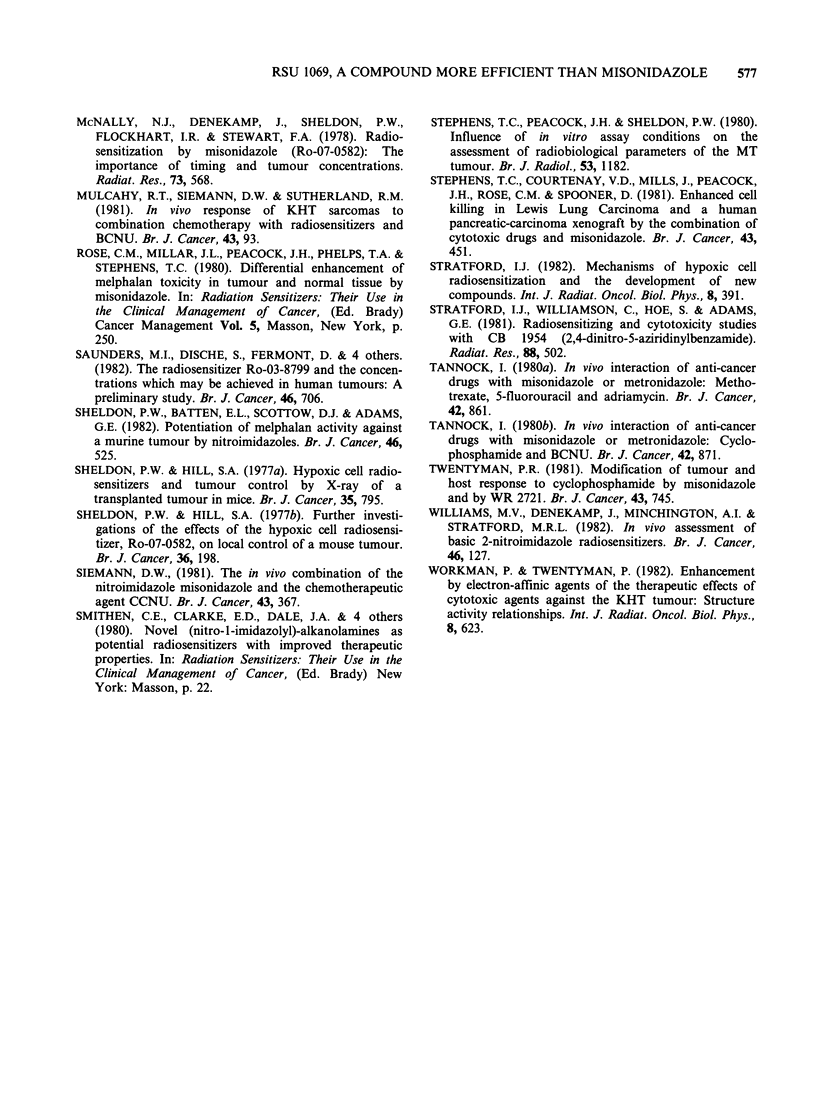# Radiation sensitization and chemopotentiation: RSU 1069, a compound more efficient than misonidazole in vitro and in vivo.

**DOI:** 10.1038/bjc.1984.91

**Published:** 1984-05

**Authors:** G. E. Adams, I. Ahmed, P. W. Sheldon, I. J. Stratford

## Abstract

Electron affinity as measured by the one-electron reduction potential, E17, is the major factor influencing radiosensitizing efficiency in vitro. RSU 1069 has an electron affinity (E17 = -398 mV) similar to misonidazole; however, the ability of this compound to sensitize hypoxic cells is considerably greater than that of misonidazole, e.g. 0.2 mM RSU 1069 gives an enhancement ratio of 2.2 compared to 1.5 for the same concentration of misonidazole. Radiosensitization studies with the MT tumour in vivo also showed RSU 1069 to be a more efficient sensitizer than misonidazole. An administered dose of only 0.08 mg g-1 RSU 1069 yielded an enhancement of 1.8 to 1.9 using tumour cell survival and tumour cure as end-points. The ability of RSU 1069 to potentiate the cytotoxic action of melphalan towards the MT tumour was also examined. RSU 1069 (0.08 mg g-1) given to mice 1 h before melphalan resulted in an enhancement of 3.0. In contrast, previous studies had shown with a series of nitroimidazoles including misonidazole that Ro 03-8799 was the most effective potentiating agent, but this only gave an enhancement of 2.3 at a 10-fold higher dose than RSU 1069. RSU 1069 is a compound of substantial promise both as a radiosensitizer and chemopotentiating agent and warrants further investigation.


*Br. J. Cancer* (1984), **49**, 571–577

### Radiation sensitization and chemopotentiation: RSU 1069, a compound more efficient than misonidazole *in vitro* and *in vivo*

G.E. Adams, I. Ahmed, P.W. Sheldon & I.J. Stratford

*Physics Department, Institute of Cancer Research, Sutton, Surrey, SM2 5PX, UK.*

**Summary** Electron affinity as measured by the one-electron reduction potential, $E^1_7$, is the major factor influencing radiosensitizing efficiency *in vitro*. RSU 1069 has an electron affinity ($E^1_7 = -398$ mV) similar to misonidazole; however, the ability of this compound to sensitize hypoxic cells is considerably greater than that of misonidazole, e.g. 0.2 mM RSU 1069 gives an enhancement ratio of 2.2 compared to 1.5 for the same concentration of misonidazole. Radiosensitization studies with the MT tumour *in vivo* also showed RSU 1069 to be a more efficient sensitizer than misonidazole. An administered dose of only 0.08 mg g$^{-1}$ RSU 1069 yielded an enhancement of 1.8 to 1.9 using tumour cell survival and tumour cure as end-points.

The ability of RSU 1069 to potentiate the cytotoxic action of melphalan towards the MT tumour was also examined. RSU 1069 (0.08 mg g$^{-1}$) given to mice 1 h before melphalan resulted in an enhancement of 3.0. In contrast, previous studies had shown with a series of nitroimidazoles including misonidazole that Ro 03-8799 was the most effective potentiating agent, but this only gave an enhancement of 2.3 at a 10-fold higher dose than RSU 1069.

RSU 1069 is a compound of substantial promise both as a radiosensitizer and chemopotentiating agent and warrants further investigation.

Many electron affinic compounds can act as hypoxic cell radiosensitizers in experimental systems. One, misonidazole (MISO), has undergone considerable clinical evaluation but it is now clear that the neurotoxic properties of this drug will seriously limit its application. This is because the maximum clinical doses that can be achieved fall well short of those required for maximum sensitizing effectiveness.

*In vitro* studies have shown that electron affinity is generally the predominant factor which determined both the cytotoxic and radiation sensitizing efficiencies of hypoxia-mediated drugs (Adams *et al.*, 1979*a*, *b*, 1980*a*, *b*). However, there are some exceptions; in particular a number of compounds which show sensitizing efficiency much greater than would be predicted from their electron affinities. One such compound is the aziridinyl dinitrobenzene, CB 1954, synthesized originally as a cytotoxic agent (Cobb *et al.*, 1969) and shown to have both alkylating and anti-metabolite properties (Connors & Malzack, 1971; Connors *et al.*, 1972). CB 1954 has an electron affinity similar to that of MISO but its ability to sensitize hypoxic cells to radiation is greater than that of MISO both *in vitro* and *in vivo* (Chapman *et al.*, 1979; Stratford *et al.*, 1981; Stratford, 1982).

The compound 2-phenyl-4(5)-amino-5(4)-carboxamide (phenyl AIC) is known to protect against the cytotoxic effects of CB 1954 (Hickman & Melzack, 1975). It has been shown that this compound, when present during irradiation, also reduces the efficiency of CB 1954 as a radiosensitizer to the level which would be predicted from its electron affinity (Stratford *et al.*, 1981). It was proposed, therefore, that the additional sensitization normally seen with CB 1954 might be associated with its cytotoxic activity.

We have subsequently included an aziridine group in a 2-nitroimidazole. The compound synthesized is RSU 1069 (NSC 347503, 1(2-nitro-1-imidazolyl)-3-aziridino-2-propanol) and its structure is given in figure 2. This agent shows considerably greater activity than MISO both *in vitro* and *in vivo*. This paper describes studies on radiosensitization by RSU 1069 and on its ability to act as a powerful potentiator of the anti-tumour action of melphalan.

### Materials and methods

### Compounds

The compound RSU 1069 was synthesized from azomycin in a similar manner to that described for other (2-nitro-1-imidazolyl)propanolamines

Present address of all authors: MRC Radiobiology Unit, Harwell, Didcot, Oxfordshire, OX11 0RD, UK.

Correspondence: G.E. Adams

Received 4 January 1984; accepted 3 February 1984.

© The Macmillan Press Ltd., 1984

(Smithen *et al.*, 1980) see Appendix. MISO and Ro 03-8799 were supplied by Dr C. Smithen, Roche Products Ltd., Welwyn Garden City, Hertfordshire, U.K. Melphalan (L-Pam, L-phenylalanine mustard) was obtained from Burroughs Wellcome, Beckenham, Kent, U.K.

*Chemical properties*

One-electron reduction potential, $E^1_7$, and the octanol:water partition coefficients, P, were measured as described previously (Adams *et al.*, 1976). The pKa of RSU 1069 was measured in aqueous media at room temperature using standard spectrophotometric methods.

*Biological studies*

Sensitizing efficiencies *in vitro* were obtained from changes in slopes of the linear portions of the radiation survival curves for hypoxic Chinese hamster V79 cells irradiated in the presence of various concentrations of RSU 1069.

Radiosensitization *in vivo* was measured using the anaplastic MT tumour implanted s.c. over the sacral region of the backs of female WHT/Cbi mice. When the tumours reached 5–6 mm in diameter, the unanaesthetised mice were dosed i.p. with RSU 1069 in saline, and locally irradiated with 230 kV X-rays (Sheldon & Hill, 1977*a*). Tumour response was determined either by an *in vitro* soft agar clonogenic assay (Sheldon *et al.*, 1982) or by the probability of local tumour control at 80 days (Sheldon & Hill, 1977*b*). The soft agar clonogenic assay was also used in the chemopotentiation studies, where male mice bearing the MT tumour were treated i.p. with $5\,mg\,kg^{-1}$ melphalan. The *in vitro* assay for tumour response was always carried out 18 h after radiation and/or drug treatment.

### Results

*Radiosensitization* in vitro

Enhancement ratios were determined for radiation sensitization of hypoxic Chinese hamster cells *in vitro* by RSU 1069 using previously described protocols (Adams *et al.*, 1976, 1979*a*, 1980*a*). At the maximum concentration tested, RSU 1069 reduced plating efficiency of unirradiated cells by no more than 20% after a 2 h contact time in hypoxia at room temperature. Full hypoxic survival curves were obtained for a range of concentrations of RSU 1069. Some examples are shown in Figure 1. At these concentrations RSU 1069 greatly increases the radiation response of hypoxic cells. In

**Figure 1** Survival curves for irradiated V79-379A cells. Sensitization by RSU 1069. Solid symbols: air; (●) control; (▲) $0.15\,mmol\,dm^{-3}$. Open symbols: nitrogen; (○) control; (□) $0.1\,mmol\,dm^{-3}$; (△) $0.2\,mmol\,dm^{-3}$.

contrast, cells irradiated in air are not sensitized by this compound. Enhancement ratios for some concentrations were obtained from a single survival point (usually between $2\times10^{-2}$ and $10^{-1}$) obtained by appropriate choice of radiation dose and by assuming an unchanged extrapolation number. Figure 2 shows the concentration dependence of the enhancement ratios for RSU 1069. The ratios for hypoxic cells irradiated in the presence of MISO (dashed line in Figure 2) were similar to those reported previously for this cell line (Adams *et al.*, 1976). Clearly RSU 1069 is a more efficient sensitizer than MISO, i.e. a lower concentration is required for any given value of ER. A comparison of the values of ER obtained at a concentration of 0.2 mM shows 2.2 for RSU 1069 and 1.5 for MISO; a concentration of 2 mM MISO is required to give an ER of 2.2.

Table I shows some physical chemical data for RSU 1069, MISO and some other compounds under consideration at the present time as sensitizers for clinical use. Also included in the table are values of sensitization efficiency ($C_{1.6}$), the concentration required to give an ER of 1.6. All compounds, with the exception of Ro 03-8799, have similar electron affinities as measured by their one-electron reduction potentials, $E^1_7$. The $E^1_7$ for Ro 03-8799 is somewhat higher and this is the only compound which *in vitro* shows sensitizing efficiency close to that for RSU 1069.

**Figure 2** Dependence of enhancement ratio for irradiated hypoxic V79-379A cells in the presence of various concentrations of RSU 1069.

**Table I** Chemical and sensitizing efficiency data for compounds of interest as potential hypoxic cell radiosensitizers for clinical use

| *Compound* | $E^1_7/mV$ | $P$[d] | *pKa* | $C_{1.6}/mol.dm^{-3}$ |
|---|---|---|---|---|
| RSU 1069 | | | | |
| (NSC 347503) | −398 | 0.22 | 6.0 | $7.5\times10^{-5}$ |
| MISO | −389[a] | 0.41[a] | — | $3.0\times10^{-4}$[a] |
| Desmethylmisonidazole | −389[a] | 0.1[a] | — | $1.0\times10^{-3}$[a] |
| Ro 03-8799 | −346[b] | 8.5[b] | 8.71 | $1.0\times10^{-4}$[b] |
| SR 2508 | −388 | 0.046[c] | — | $3.0\times10^{-4}$ |

[a]Adams *et al.*, 1976.
[b]Smithen *et al.*, 1980.
[c]Brown & Workman 1980.
[d]*P* values for RSU 1069 and Ro 03-8799 were determined for the unprotonated bases (i.e. pH > 11.0).

*In* vivo *studies*

*Toxicity* The $LD_{50/7}$ for RSU 1069 administered i.p. in saline to female WHT/Cbi mice is $0.15\,mg\,g^{-1}$. At the $LD_{50}$ dose, death occurred 4 to 6 days after treatment. In contrast, for compounds such as MISO, that cause neurological damage, death occurred within a day of dosing. This probably indicates that the dose limiting tissue for RSU 1069 lethality is not the same as that for misonidazole.

*Radiosensitization* It is known that, due to pharmacokinetic considerations, the time at which a sensitizer is administered to tumour-bearing mice prior to irradiation can greatly influence the observed radiation response (Sheldon & Hill, 1977*b*; McNally *et al.*, 1978; Brown & Yu, 1980). Therefore in initial experiments RSU 1069 was given i.p. to tumour-bearing mice at various times before an X-ray dose of 17 Gy. The response of the MT tumour to radiation $+0.08\,mg\,g^{-1}$ RSU 1069 assessed by a clonogenic assay technique is shown in Figure 3. Irradiation with a dose of 17 Gy alone only reduces tumour cell survival to $2\times10^{-2}$. This is in line with previous data (Stephens *et al.*, 1980) indicating that this tumour contains radiation-resistant hypoxic cells. The decrease in cell survival due to sensitization is dependent upon the interval between drug administration and irradiation. Maximum sensitization appears to occur when the drug is given no earlier than 90 min before commencement of irradiation. For this time range survival is decreased to around $3\times10^{-4}$. When RSU 1069 is given without irradiation, tumour cell survival is reduced to 0.5. Taking this into account, the maximum sensitization for this drug dose corresponds to an enhancement ratio of 1.8. This level of radiosensitization with MISO, in this tumour system, requires at least a 15-fold higher dose (Adams *et al.*, 1984).

The sensitization efficiency of RSU 1069 was also measured *in vivo* by the $TCD_{50}$ method. A single dose of $0.08\,mg\,g^{-1}$ was administered 90 min before the mice were given a range of doses of X-irradiation in order to assess the probability of local control of the MT tumour at 80 days. Data are shown in Figure 4. In the absence of drug, 68 Gy were required to achieve a 50% probability of tumour control ($TCD_{50}$). In the drug-tested mice, the value of the $TCD_{50}$ was reduced to 35 Gy. This corresponds to an enhancement ratio of 1.9, and is consistent with the ER obtained from the clonogenic assay.

*Chemopotentiation* We have recently reported chemopotentiation by misonidazole and a variety of other nitroimidazoles of melphalan damage in the

**Figure 3** Radiosensitization of the MT tumour in WHT mice by RSU 1069. Effect of time between drug administration and irradiation. Each point is derived from 2 to 4 pooled tumours.

**Figure 4** Probability of local control of the MT tumour in WHT mice by radiation alone (X) or by radiation plus $0.08\,mg\,g^{-1}$ RSU 1069 given i.p. to mice 90 min prior to X-rays (O). The standard errors on the values of $TCD_{50}$ are illustrated by the horizontal bars. Eight mice were treated per point.

MT tumour implanted intramuscularly (Sheldon *et al.*, 1982). This study showed that the compound Ro 03-8799 produced the greatest potentiation. We have compared the chemopotentiating properties of RSU 1069 with Ro 03-8799 using the clonogenic assay endpoint for subcutaneous tumours. Figure 5 shows the tumour cell response when the nitroimidazoles were given to mice before or after $5\,mg\,kg^{-1}$ melphalan. Melphalan alone reduces survival to $5 \times 10^{-2}$ and the nitroimidazoles alone reduce survival by no more than 50%. However, the combination of the nitroimidazole with melphalan can cause a large increase in cell killing. The amount of potentiation is dependent upon the timing and sequencing of the drug combination. For Ro 03-8799 the optimum time is 30 min. Before melphalan, whereas for RSU 1069 it is 60 min. A dose of $0.72\,mg\,g^{-1}$ Ro 03-8799 with melphalan produced a maximum reduction in cell survival of $10^{-3}$, whereas a 10-fold *lower* dose of RSU 1069 caused a further 10-fold decrease in cell survival. These survival levels correspond to enhancement ratios of 2.3 for Ro 03-8799 and 3.0 for RSU 1069.

**Figure 5** Potentiation of the cytotoxic effect of melphalan towards the MT tumour in WHT mice. The effects of Ro 03-8799 (X) or RSU 1069 (O) given at various times before or after $0.5\,\mu g\,g^{-1}$ melphalan. Each point is derived from 2 to 4 pooled tumours.

### Discussion

Some compounds (Desmethylmisonidazole, SR 2508 and Ro 03-8799) have been or are being considered as successors to misonidazole for clinical use. Desmethylmisonidazole and SR 2508 have similar sensitizing efficiencies to MISO both *in vitro* and *in vivo*. They were chosen on the basis of pharmacokinetic considerations suggesting the drugs would be less neurotoxic (Dische *et al.*, 1980; Brown *et al.*, 1981). Ro 03-8799 showed greater sensitizing efficiency than MISO *in vitro*, but this was not reflected *in vivo*, where levels of sensitization were obtained which were no greater than would be expected from a similar administered dose of MISO (Williams *et al.*, 1982). However, the neurotoxicity of Ro 03-8799 was lower than MISO in baboons, (Eichler & Jackson personal communication, in Saunders *et al.*, 1982).

The compound RSU 1069 shows substantially greater radiosensitizing efficiency than MISO both *in vitro* and *in vivo*. This is the first nitroimidazole shown to be substantially more efficient than MISO *in vivo*. It warrants further evaluation in other tumour systems using both single and multi-fraction radiation regimes. It should be pointed out, that RSU 1069 is more toxic than MISO as measured by acute $LD_{50}$ values in WHT/Cbi female mice where the respective $LD_{50}$'s are $0.15\,mg\,g^{-1}$ and $1.8\,mg\,g^{-1}$. However, it would be premature at this stage to make any conclusions regarding relative therapeutic ratios without more detailed toxicological evaluation.

There is currently considerable interest in the use of MISO and other nitroimidazoles as potentiators of alkylating agents *in vivo* (Rose *et al.*, 1980; Clement *et al.*, 1980 Tannock, 1980*a*, *b*; Law *et al.*, 1981; Martin *et al.*, 1981; Siemann, 1981; Mulcahy *et al.*, 1981; Stephens *et al.*, 1981; Twentyman, 1981). Most of these combination studies have been concerned with MISO. Recently it has been shown that other nitro compounds can show potentiating activity considerably greater than MISO (Workman & Twentyman, 1982; Sheldon *et al.*, 1982). In one of these studies (Sheldon *et al.*, 1982) Ro 03-8799 was found to be the most effective potentiator of melphalan damage in the MT tumour, when compared with a range of other nitroimidazoles including MISO. RSU 1069 shows even greater activity than Ro 03-8799.

If there were to be a wide role for hypoxic cell sensitizers in radiotherapy, agents more effective than MISO are required. There may also be a clinical use for nitroimidazoles as chemopotentiators. On the basis of the results reported here, the compound RSU 1069 merits further investigation

The Drug Synthesis and Development Branch kindly provided azomycin, which was used in the synthesis of RSU 1069. We gratefully acknowledge the excellent technical assistance given to us by Christine Williamson and Dev Rakshit; we thank also Drs Peter O'Neill and Stephen Hoe for the determination of the value of $E_7^1$ for RSU 1069 and SR 2508.

This work was supported by grants from the MRC and NCI (contract No. 1-CH-17502).

### Appendix

RSU 1069 gave satisfactory elemental and mass spectural analyses. $^1$H.n.m.r. spectra were recorded in $CDCl_3$ relative to 2,2-dimethyl-2-silopentane-5-sulphonate as internal standard. Spin multiplicities are indicated by the symbols s(singlet), m (multiplet). Infrared spectra were recorded in nujol.

1-(2,3-epoxypropyl)-2-nitroimidazole (RSU 1062) was prepared using the method described by Beaman *et al.* (1967). RSU 1062 was mixed with a twice molar excess of aziridine in methanol and heated under reflux for one hour. The solvent was removed under reduced pressure and the residue recrystallised from ethanol to give RSU 1069, mp 119–121°C, as a pale yellow crystalline solid (yield 56%). I.R.$\nu$(cm$^{-1}$): 3160, 3120, 3080, 1530, 1500, 1483, 1290, 1262, 1157, 1140, 1007, 920, 887, 848, 836, 787, 775, 650 and 630. $^1$H n.m.r. $\delta$(p.p.m.): 7.35 (d,l,imid.*H*), 7.08 (d,l,imid.*H*), 4.15–5.01 (m,4,imid-$CH_2$-CHOH-, peak at 4.51 exchanging with $D_2O$), 2.32 (m,2,$CH_2$-aziridine), 1.67 (m,2, aziridine) and 1.21 (m,2,aziridine).

### References

ADAMS, G.E., AHMED, I., CLARKE, E.D. & 7 others. (1980*a*). Structure-activity relationships in the development of hypoxic cell radiosensitizers III. Effects of basic substituents in nitroimidazole sidechains. *Int. J. Radiat. Biol.*, **38**, 613.

ADAMS, G.E., AHMED, I., SHELDON, P.W. & STRATFORD, I.J. (1984). RSU 1069, a 2-nitroimidazole containing an alkylating group: High efficiency as a radio- and chemosensitizer *in vitro* and *in vivo*. *Int. J. Radiat. Oncol. Biol. Phys.* (In press).

ADAMS, G.E., CLARKE, E.G., FLOCKHART, I.R. & 8 others. (1979*a*). Structure-activity relationships in the development of hypoxic cell radiosensitizers I. Sensitizing efficiency. *Int. J. Radiat. Biol.*, **35**, 133.

ADAMS, G.E., CLARKE, E.D., GRAY, P. & 7 others. (1979*b*). Structure-activity relationships in the development of hypoxic cell radiosensitizers II. Cytotoxicity and therapeutic ratio. *Int. J. Radiat. Biol.*, **35**, 151.

ADAMS, G.E., FLOCKHART, I.R., SMITHEN, C.E., STRATFORD, I.J., WARDMAN, P. & WATTS, M.E. (1976). Electron-affinic sensitization VII. A correlation between structure, one-electron reduction potentials and efficiencies of nitroimidazoles as hypoxic cell radiosensitizers. *Radiat. Res.*, **67**, 9.

ADAMS, G.E., STRATFORD, I.J., WALLACE, R.G., WARDMAN, P. & WATTS, M.E. (1980*b*). Toxicity of nitro compounds toward hypoxic mammalian cells: Dependence upon reduction potential. *J. Natl Cancer Inst.*, **64**, 555.

BEAMAN, A.G., TAUTZ, W. & DUSCHINSKY, R. (1967). Studies in the Nitroimidazole Series: III. 2-Nitroimidazole derivitives substituted in chi-position. *Antimicrobial Agents Chemother.*, 1968, 520.

BROWN, J.M. & WORKMAN, P. (1980). Partition coefficient as a guide to the development of radiosensitizers which are less toxic than misonidazole. *Radiat. Res.*, **82**, 171.

BROWN, J.M. & YU, N.Y. (1980). The optimum time for irradiation relative to tumour concentration of hypoxic cell sensitizers. *Br. J. Radiol.*, **53**, 915.

BROWN, J.M., YU, N.Y., BROWN, D.M. & LEE, W. (1981). SR 2508: A 2-nitroimidazole amide which should be superior to misonidazole as a radiosensitizer for clinical use. *Int. J. Radiat. Oncol. Biol. Phys.*, **7**, 695.

CHAPMAN, J.D., RALEIGH, J.A., PEDERSON, J.E. & 4 others. (1979). Potentially three distinct roles for hypoxic cell sensitizers in the clinic. *Proc. 6th Int. Cong. Radiation Research*, Tokyo, Japan, 1979. (Eds. Okada *et al.*) (JARR, Tokyo), pp. 885–892.

CLEMENT, J.J., GORMAN, M.S., WODINSKY, I., CATANE, R. & JOHNSON, R.K. (1980). Enhancement of anti-tumour activity of alkylating agents by the radiation sensitizer misonidazole. *Cancer Res.*, **40**, 4165.

COBB, L.M., CONNORS, T.A., ELSON, L.A. & 4 others. (1969). 2,4-dinitro-5-ethyleneiminobenzamide (CB1954): A potent and selective inhibitor of the growth of the Walker carcinoma 256. *Biochem. Pharmacol.*, **18**, 1519.

CONNORS, T.A., MANDEL, H.G. & MELZAK, D.H. (1972). Studies on the reversal of the selective anti-tumour effect of the aziridinyl derivative CB 1954 by 4-amino-5-imidazolecarboxamide. *Int. J. Cancer*, **9**, 126.

CONNORS, T.A. & MELZACK, D.J. (1971). Studies on the mechanism of action of 5-aziridimyl-2,4-dinitrobenzamide (CB 1954), a selective inhibitor of the Walker tumour. *Int. J. Cancer*, **7**, 86.

DISCHE, S., FOWLER, J.F., SAUNDERS, M.I. & 4 others. (1980). Desmethylmisonidazole, a drug for improved radiosensitization on radiotherapy. *Br. J. Cancer*, **42**, 153.

HICKMAN, J.A. & MELZACK, D.H. (1975). Protection against the effects of the antitumour agent CB 1954 by certain imidazoles and related compounds. *Biochem. Pharmacol.*, **24**, 1947.

LAW, M.P., HIRST, D.G. & BROWN, J.M. (1981). The enhancing effect of misonidazole on the response of the RIFI tumour to cyclophosphamide. *Br. J. Cancer*, **44**, 208.

MARTIN, W.M.C., McNALLY, N.J. & deRONDE, J. (1981). Enhancement of the effect of cytotoxic drugs by radiosensitizers. *Br. J. Cancer*, **43**, 756.

McNALLY, N.J., DENEKAMP, J., SHELDON, P.W., FLOCKHART, I.R. & STEWART, F.A. (1978). Radiosensitization by misonidazole (Ro-07-0582): The importance of timing and tumour concentrations. *Radiat. Res.*, **73**, 568.

MULCAHY, R.T., SIEMANN, D.W. & SUTHERLAND, R.M. (1981). *In vivo* response of KHT sarcomas to combination chemotherapy with radiosensitizers and BCNU. *Br. J. Cancer*, **43**, 93.

ROSE, C.M., MILLAR, J.L., PEACOCK, J.H., PHELPS, T.A. & STEPHENS, T.C. (1980). Differential enhancement of melphalan toxicity in tumour and normal tissue by misonidazole. In: *Radiation Sensitizers: Their Use in the Clinical Management of Cancer*, (Ed. Brady) Cancer Management **Vol. 5**, Masson, New York, p. 250.

SAUNDERS, M.I., DISCHE, S., FERMONT, D. & 4 others. (1982). The radiosensitizer Ro-03-8799 and the concentrations which may be achieved in human tumours: A preliminary study. *Br. J. Cancer*, **46**, 706.

SHELDON, P.W., BATTEN, E.L., SCOTTOW, D.J. & ADAMS, G.E. (1982). Potentiation of melphalan activity against a murine tumour by nitroimidazoles. *Br. J. Cancer*, **46**, 525.

SHELDON, P.W. & HILL, S.A. (1977*a*). Hypoxic cell radiosensitizers and tumour control by X-ray of a transplanted tumour in mice. *Br. J. Cancer*, **35**, 795.

SHELDON, P.W. & HILL, S.A. (1977*b*). Further investigations of the effects of the hypoxic cell radiosensitizer, Ro-07-0582, on local control of a mouse tumour. *Br. J. Cancer*, **36**, 198.

SIEMANN, D.W., (1981). The *in vivo* combination of the nitroimidazole misonidazole and the chemotherapeutic agent CCNU. *Br. J. Cancer*, **43**, 367.

SMITHEN, C.E., CLARKE, E.D., DALE, J.A. & 4 others (1980). Novel (nitro-1-imidazolyl)-alkanolamines as potential radiosensitizers with improved therapeutic properties. In: *Radiation Sensitizers: Their Use in the Clinical Management of Cancer*, (Ed. Brady) New York: Masson, p. 22.

STEPHENS, T.C., PEACOCK, J.H. & SHELDON, P.W. (1980). Influence of *in vitro* assay conditions on the assessment of radiobiological parameters of the MT tumour. *Br. J. Radiol.*, **53**, 1182.

STEPHENS, T.C., COURTENAY, V.D., MILLS, J., PEACOCK, J.H., ROSE, C.M. & SPOONER, D. (1981). Enhanced cell killing in Lewis Lung Carcinoma and a human pancreatic-carcinoma xenograft by the combination of cytotoxic drugs and misonidazole. *Br. J. Cancer*, **43**, 451.

STRATFORD, I.J. (1982). Mechanisms of hypoxic cell radiosensitization and the development of new compounds. *Int. J. Radiat. Oncol. Biol. Phys.*, **8**, 391.

STRATFORD, I.J., WILLIAMSON, C., HOE, S. & ADAMS, G.E. (1981). Radiosensitizing and cytotoxicity studies with CB 1954 (2,4-dinitro-5-aziridinylbenzamide). *Radiat. Res.*, **88**, 502.

TANNOCK, I. (1980*a*). *In vivo* interaction of anti-cancer drugs with misonidazole or metronidazole: Methotrexate, 5-fluorouracil and adriamycin. *Br. J. Cancer*, **42**, 861.

TANNOCK, I. (1980*b*). *In vivo* interaction of anti-cancer drugs with misonidazole or metronidazole: Cyclophosphamide and BCNU. *Br. J. Cancer*, **42**, 871.

TWENTYMAN, P.R. (1981). Modification of tumour and host response to cyclophosphamide by misonidazole and by WR 2721. *Br. J. Cancer*, **43**, 745.

WILLIAMS, M.V., DENEKAMP, J., MINCHINGTON, A.I. & STRATFORD, M.R.L. (1982). *In vivo* assessment of basic 2-nitroimidazole radiosensitizers. *Br. J. Cancer*, **46**, 127.

WORKMAN, P. & TWENTYMAN, P. (1982). Enhancement by electron-affinic agents of the therapeutic effects of cytotoxic agents against the KHT tumour: Structure activity relationships. *Int. J. Radiat. Oncol. Biol. Phys.*, **8**, 623.